# The Bright, the Dark, and the Blue Face of Narcissism: The Spectrum of Narcissism in Its Relations to the Metatraits of Personality, Self-Esteem, and the Nomological Network of Shyness, Loneliness, and Empathy

**DOI:** 10.3389/fpsyg.2018.00343

**Published:** 2018-03-14

**Authors:** Radosław Rogoza, Magdalena Żemojtel-Piotrowska, Maria M. Kwiatkowska, Katarzyna Kwiatkowska

**Affiliations:** Institute of Psychology, Cardinal Stefan Wyszyński University in Warsaw, Warsaw, Poland

**Keywords:** vulnerable narcissism, rivalry, admiration, personality, self-esteem

## Abstract

Grandiose and vulnerable narcissism seem to be uncorrelated in empirical studies, yet they share at least some theoretical similarities. In the current study, we examine the relation between grandiose (conceptualized as admiration and rivalry) and vulnerable narcissism in the context of the Big Five personality traits and metatraits, self-esteem, and their nomological network. To this end, participants (*N* = 314) filled in a set of self-report measures via an online survey. Rivalry was positively linked with both admiration and vulnerable narcissism. We replicated the relations of admiration and rivalry with personality traits and metatraits—as well as extended existing knowledge by providing support for the theory that vulnerable narcissism is simultaneously negatively related to the Stability and Plasticity. Higher scores on vulnerable narcissism and rivalry predicted having fragile self-esteem, whereas high scores on admiration predicted having optimal self-esteem. The assumed relations with the nomological network were confirmed, i.e., vulnerable narcissism and admiration demonstrated a contradictory pattern of relation to shyness and loneliness, whilst rivalry predicted low empathy. Our results suggest that the rivalry is between vulnerable narcissism and admiration, which supports its localization in the self-importance dimension of the narcissism spectrum model. It was concluded that whereas admiration and rivalry represent the bright and dark face of narcissism, vulnerable narcissism represents its blue face.

## Introduction

Narcissism can be described as a construct with different faces used for different purposes, which are shown depending on the situational assessment (Rogoza, in press). The word “narcissism” in common use describes someone who is excessively self-absorbed, selfish and egoistical, self-enhancing, arrogant and shameless (Jonason et al., 2012); however, from a scientific perspective, two forms of narcissism can be distinguished: vulnerable and grandiose narcissism (Wink, 1991; Pincus et al., 2009; Miller et al., 2012). This distinction causes controversies to emerge as despite both being called narcissism, they are heterogenous constructs (Miller et al., 2017a). According to the research tradition, vulnerable narcissism has most often been interpreted as clinical narcissism due to its intra- and inter-personally malevolent correlates such as hypersensitivity, introversion, shyness, vulnerability to depression, incompetence, anxiety, defensiveness, avoidance, hostility, passive aggression, low self-esteem, and poor well-being (Wink, 1991; Hendin and Cheek, 1997; Rose, 2002; Dickinson and Pincus, 2003; Miller et al., 2011; Brown et al., 2016). The central features of vulnerable narcissism as rated by the experts are negative temperament, neuroticism, borderline traits, mistrust, depression, and anxiety (Thomas et al., 2012; Miller et al., 2017a). Grandiose narcissism was most often interpreted as normal or subclinical narcissism because it encompasses a blend of correlates that are both positive—e.g., assertiveness, self-confidence, self-efficacy, charm, extraversion, high self-esteem, and well-being—and negative—e.g., aggressiveness, antagonism, dominance, disagreeableness, entitlement, and exploitativeness (Morf and Rhodewalt, 2001; Rose, 2002; Ackerman et al., 2011; Miller et al., 2011; Back et al., 2013; Brookes, 2015; Krizan and Herlache, 2017). The descriptions of expert ratings suggest that central features of grandiose narcissism are treatment rejecting, manipulativeness, entitlement, exhibitionism, antisociality, and low agreeableness (Thomas et al., 2012; Miller et al., 2017a). Although grandiose and vulnerable narcissism are uncorrelated with each other (Wink, 1991; Hendin and Cheek, 1997), they share some common characteristics such as self-importance, aggressiveness, grandiose fantasies, lack of empathy, entitlement, and low communion (Pincus et al., 2009; Miller et al., 2011, 2012; Brown et al., 2016; Krizan and Herlache, 2017). Given the lack of correlation, but at the same time similarities in characteristics of the two forms of narcissism, the potential sources of their (lack of) relationship seems to be an important research topic. We posit that a possible explanation of the dissimilarities and similarities between grandiose and vulnerable narcissism is to be found in an exploration of the so called bright and dark side of grandiose narcissism (Back et al., 2013).

### Grandiose Narcissism

Back et al. (2013) noted that the research on narcissism advanced over the years but still was influenced by the existence of the Narcissistic Personality Inventory (NPI, Raskin and Hall, 1979); thus, they proposed a new conceptualization that integrated existing knowledge on grandiose narcissism and disentangled some of the actual paradoxes. Within the new conceptualization of grandiose narcissism—i.e., the Narcissistic Admiration and Rivalry Concept (NARC)—two distinct but related dimensions exist that serve the same goal (i.e., maintenance of the grandiose self) via different means (Back et al., 2013). The first dimension, narcissistic admiration, represents the self-enhancing and assertive aspect of narcissism. This dimension encompasses grandiose fantasies, striving for uniqueness, and self-promoting behaviors that aim for social admiration and boost the narcissistic ego. The second dimension, narcissistic rivalry, represents the self-defensive and antagonistic aspect of narcissism. It comprises devaluation of others, striving for supremacy, and hostile behaviors that aim to diminish the threat to the ego and may result in social conflicts (Back et al., 2013). Narcissistic admiration is associated primarily with positive correlates such as high self-esteem, grandiosity, benign envy, gratitude, forgiveness, and lower distrust, while their motivation focuses on achievements, self-direction, hedonism, and stimulation; narcissistic rivalry, however, is primarily associated with negative correlates such as low self-esteem, impulsivity, malicious envy, loneliness, low empathy, low trust, and lack of forgiveness, and it is driven by the motive of power (Back et al., 2013; Lange et al., 2016; Rogoza et al., 2016b; Wetzel et al., 2016; Fatfouta, 2017; Fatfouta and Schröder-Abé, 2017; Geukes et al., 2017). The contradictory relations with some of the variables should, however, be interpreted with caution resulting from the high social desirability of narcissists (Kowalski et al., 2018)—for example forgiveness, while studied using implicit measures, is unrelated either to admiration or rivalry (Fatfouta et al., 2017). This differentiation is also reflected in the social functioning of grandiose narcissists; Paulhus (1998) noted that they are initially perceived as charming, competent, and popular, but this perception decreases significantly over time. The NARC model supplemented this picture, as narcissists at the moment of zero acquaintance are seen as charming (Back et al., 2010) due to the positive impact of the admiration dimension, but the negative impact of rivalry undermines these judgements over time (Leckelt et al., 2015) and reveals the exploitative nature of grandiose narcissism (Morf and Rhodewalt, 2001).

### Vulnerable Narcissism

As a more clinical expression of narcissism (Cain et al., 2008), the vulnerable form, in addition to self-absorbance and entitlement, represents pathological psychological distress and fragility (Miller et al., 2017a). One of the popular measures of vulnerable narcissism, the Hypersensitive Narcissism Scale (HSNS, Hendin and Cheek, 1997), was developed on the basis of Murray’s narcissism scale (Murray, 1938), in which narcissism was interpreted as indicating dual dynamics: on the one hand, it was related to self-enhancement, exploitation, and craving for attention (i.e., reflecting grandiose narcissism), while on the other, it was positively related to feelings of being neglected and belittled, hypersensitivity, and anxiety (i.e., reflecting vulnerable narcissism). The HSNS was derived from items that were capturing the meaning of vulnerable narcissism—statements that were selected on the basis of correlations with the composite MMPI-based (i.e., concerning narcissistic personality disorder) narcissism scale. Ten items that were the most diagnostic regarded hypersensitivity and vulnerability as conceptualized by Wink (1991), and significantly loaded on a single factor in three different samples (Hendin and Cheek, 1997). Thus, the HSNS could be considered as a general unidimensional measure of vulnerable narcissism.

Apart from the HSNS (Hendin and Cheek, 1997), other measures of vulnerable narcissism also exist, such as the Pathological Narcissism Inventory (PNI; Pincus et al., 2009) and the Five Factor Narcissism Inventory (FFNI; Glover et al., 2012). Whereas the PNI originated from clinical psychology, the FFNI was developed in personality psychology (Pincus and Lukowitsky, 2010; Miller et al., 2016). Since both of these scales are multidimensional in nature and provide useful insight into better understanding of vulnerable narcissism through the assessment of its different components, but conceptualize narcissism differently, there is controversy as to which of these two measures best captures the phenomenon (Miller et al., 2014; Wright, 2016), and perhaps they both measure important aspects of it. However, as assessed by expert ratings, the HSNS has a similar pattern of relations with both personality traits within the normal range and pathological personality traits to the vulnerability scales from the PNI and FFNI (Miller et al., 2014); thus, it might be deemed a general marker of narcissistic vulnerability. The PNI, FFNI, and HSNS are similar measures if one considers only the general score; however, the PNI and FFNI provide a much broader perspective owing to their dimensions. That is useful in clinical practice; conversely, the HSNS is much shorter to administer, making it more useful in screening studies.

### Narcissism Spectrum Model

**FIGURE 1:**
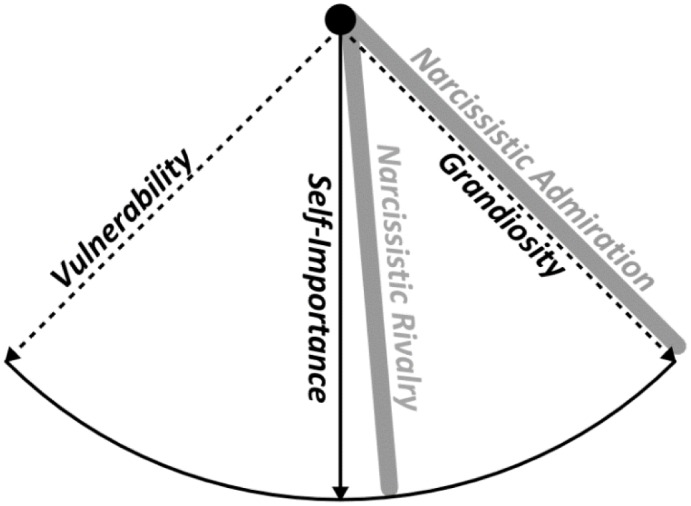
The graphical representation of the Narcissism Spectrum Model in which admiration and rivalry were assigned to the hypothesized dimensions (Krizan and Herlache, 2017).

Although grandiose and vulnerable narcissism are uncorrelated in empirical research, there is some evidence that the rivalry captures a modest amount of vulnerability (Miller et al., 2014), which can be explained within the framework of the Narcissism Spectrum Model (NSM; Krizan and Herlache, 2017), depicted in **Figure 1**.

The NSM defines the model of narcissism as a three-dimensional construct—the central part, representing the core feature of narcissism, is the self-importance and entitlement. Two other features of narcissism, grandiosity and vulnerability, diverge from self-importance in opposite directions at an angle of (almost) 90°, which makes them (almost) orthogonal (and thus – uncorrelated). The smaller the angle becomes, the stronger and more positive the relationship becomes, i.e., as the angle of the self-importance dimension equals approximately 45°, it should be moderately positively linked to both the vulnerability and grandiosity dimensions. Simultaneously, the larger the angle becomes, the stronger but more negative the relationship becomes, i.e., as the spectrum can expand even beyond the dimension of vulnerability and grandiosity, their relation may be negative. This expected pattern of relationships was partially supported in previous research, as rivalry indeed turned out to be positively related to vulnerability and grandiosity (Back et al., 2013; Miller et al., 2014). The thesis of the common core of narcissism presented in NSM is elegant, as the distinction of self-importance provides the justification to theoretically label two orthogonal constructs under the same name. Because rivalry captures such elements of the self-importance dimension as supremacy, devaluing, and aggressiveness (Back et al., 2013), owing to the logic of the spectrum model (Krizan and Herlache, 2017), it should be simultaneously related to vulnerability and grandiosity dimensions. In the current study, we aim to test this assumption through examination of the relations between these different faces of narcissism in the context of personality traits and metatraits, self-esteem, and the nomological network of shyness, loneliness, and empathy.

### Different Faces of Narcissism and Personality

#### The Basic Traits of Personality

The relationship between grandiose and vulnerable narcissism and basic personality traits has been widely examined (Hendin and Cheek, 1997; Miller et al., 2010; Ackerman et al., 2011; Houlcroft et al., 2012; Rogoza et al., 2016b). Grandiose narcissism has often been described as a mix of extraversion and low agreeableness (Paulhus, 2001). However, the study of Back et al. (2013) provided evidence that the observed relationship between grandiose narcissism and these two personality traits is rather due to two narcissistic strategies: only admiration was related to extraversion, and only rivalry was related to low agreeableness, which was also replicated in other studies (Leckelt et al., 2015; Rogoza et al., 2016b). The existing literature on vulnerable narcissism provided strong evidence that it is linked mostly to high neuroticism (Miller et al., 2017b), but other studies also suggested its relation to low agreeableness and low extraversion (Hendin and Cheek, 1997; Miller et al., 2010; Houlcroft et al., 2012; Campbell and Miller, 2013; Miller et al., 2017a). Thus, the personality characteristics of the different faces of narcissism seems to suggest that the trait that distinguishes the vulnerability and grandiosity dimensions is neuroticism (present only in vulnerable narcissism), there is an overall difference in extraversion (low in vulnerable and high in grandiose narcissism), and the two forms of narcissism have low agreeableness in common.

#### The Metatraits of Personality

The metatraits of personality, which are described in the Two Factor Model of personality, are theoretical constructs, describing the two most basic mechanisms designed to meet the need to engage in exploration of novel environments (Plasticity metatrait) and the need to maintain stability of ongoing goal-directed functioning (Stability metatrait; DeYoung et al., 2002; Cieciuch and Strus, 2017). The metatraits are the broadest dimensions of the personality hierarchical structure, which means that their influence is the most fundamental for ongoing personality functioning (DeYoung, 2015). Plasticity (which is a combination of the common variance of extraversion and openness to experience) reflects dynamism and the extent of active engagement in one’s inner (in the form of rich imagination and fantasies) and outer (in the form of behavioral exploration) worlds, whereas Stability (which is a combination of the common variance of agreeableness, conscientiousness and reversed neuroticism) reflects self-regulation, ability to realize long-term goals and the tendency to be well-socialized (Olson, 2005; Saucier et al., 2014; DeYoung, 2015; Cieciuch and Strus, 2017).

**FIGURE 2:**
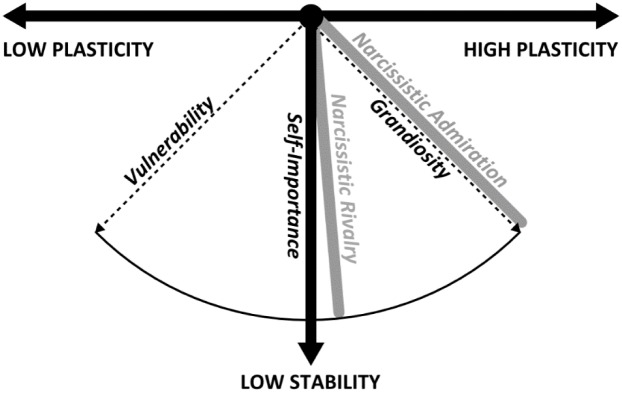
Hypothesized relations between different faces of narcissism and personality metatraits.

Rogoza et al. (2016c) investigated the relationship between grandiose narcissism and personality metatraits and reported that admiration was positively linked primarily with Plasticity, whereas rivalry was negatively linked primarily with Stability. Thus, in grandiose narcissism, Plasticity is believed to be the beneficial aspect that enables quick adaptation to social environments that vary due to an unstable pattern of relationships (Rogoza et al., 2016c). Although vulnerable narcissism was not analyzed in the context of personality metatraits, on the basis of the relation with basic traits one can expect that it may be related to both the Stability and Plasticity metatraits (Cieciuch and Strus, 2017). However, contrary to admiration, vulnerable narcissism may be negatively related to Plasticity, and similarly to rivalry, vulnerable narcissism should also be negatively related to Stability. The hypothesized relations of the different faces of narcissism with personality metatraits are illustrated on **Figure 2**.

Thus, low Stability is hypothesized to reflect the core dimension of narcissism—self-importance (Krizan and Herlache, 2017)—whereas Plasticity is expected to be the foundation of the NSM which represents both vulnerability (low) and grandiosity (high) dimensions. In this vein, the rivalry—as assumed by the NSM (Krizan and Herlache, 2017) and supported by the observed relation with low Stability (Rogoza et al., 2016c)—is expected to be located near the core of narcissism.

### Narcissism and Self-Esteem

The issue of self-esteem is one of the most fundamental within the research on narcissism (Brummelman et al., 2016). Numerous studies have reported that grandiose narcissism is moderately related to self-esteem (Pincus et al., 2009; Miller et al., 2010; Okada, 2010); however, this may again be interpreted as a suppression effect, since admiration is related stably and positively to self-esteem, while rivalry is related varyingly and negatively to it (Back et al., 2013; Rogoza et al., 2016c; Geukes et al., 2017). Similarly, vulnerable narcissism is strongly negatively related to self-esteem (Rose, 2002; Zeigler-Hill et al., 2008; Miller et al., 2010; Okada, 2010). Although contingent self-esteem (i.e., feelings about oneself that are dependent on self- and other-based disapproval; Kernis, 2003) is an important component of vulnerable narcissism (Pincus et al., 2009; Rubinstein, 2010), only a few studies investigated its relationship to grandiose narcissism. Zeigler-Hill et al. (2008) reported that vulnerable narcissism was related to all domains of contingent self-esteem (i.e., physical appearance, competition, academic competence, others’ approval, family support, god’s love, and virtue), whereas grandiose narcissism was only related to two domains—positively to competition and negatively to others’ approval.

These results suggest that the self-esteem of vulnerable narcissists is low, and their fragile sense of self-worth is hypersensitive and labile, while grandiose narcissists have generally higher and more stable self-esteem. The results concerning grandiose narcissism refer to the grandiosity dimension, while little is known about the self-importance dimension; therefore, the differentiation of admiration and rivalry may shed new light on the associations between contingent self-esteem and grandiose narcissism.

Kernis (2003) defined optimal self-esteem as a stable, authentic feeling of self-worth, with a relative absence of defensiveness and an excessively strong desire to be liked by others, and which is not dependent upon specific correlates. In other words, self-esteem is optimal when an individual’s self-esteem is high and contingent self-esteem is low. This conceptualization is similar to the categorical diagnosis of mental health, in which individuals could be described as languishing (a state of being mentally unhealthy) or as flourishing in life (a state of being mentally healthy; Keyes, 2002; Karaś et al., 2014). Because vulnerable narcissism is positively related to contingent self-esteem and negatively to self-esteem, while admiration is negatively related to contingent self-esteem and positively to self-esteem, one could hypothesize that vulnerable narcissism is more present among individuals whose self-esteem is fragile, while admiration is more present in individuals whose self-esteem is optimal.

### The Current Study

The current paper aims to investigate the narcissism spectrum, in the context of personality, self-esteem, and its nomological network. As suggested by the NSM (Krizan and Herlache, 2017), our general expectation assumes that self-importance (rivalry) is salient in both the grandiose and vulnerable dimensions of narcissism. To test this general expectation, we formulated a set of hypotheses: (1) as a preliminary direct assessment, we expected that rivalry will be positively related to both admiration and vulnerable narcissism; (2) in regard to the relation with personality, we hypothesized that rivalry would be related mostly to low agreeableness, and admiration with high extraversion, whereas vulnerable narcissism would be related mostly to high neuroticism, low extraversion and low agreeableness; (3) we hypothesized that both rivalry and vulnerable narcissism would be related to low Stability and also expected that while admiration would be positively related, vulnerable narcissism would be negatively related to Plasticity; (4) in the context of self-esteem, we hypothesized that vulnerable narcissism is more characteristic of individuals with fragile self-esteem and admiration is more characteristic of individuals with optimal self-esteem; and (5) in studying the nomological network, we hypothesized that vulnerable narcissism and admiration would have a contradictory relationship with shyness and loneliness (positive for vulnerable narcissism and negative for admiration), whereas rivalry is expected to be a negative predictor of empathy.

The first hypothesis was tested using a two-tailed significance test of the Pearson’s correlation coefficient. The second and third hypotheses were tested using linear regression models in order to control for the shared variance in which personality traits and metatraits predicted narcissism. The fourth hypothesis was tested using latent class regressions in which different faces of narcissism were tested as predictors of latent class membership. The last hypothesis was tested using linear regression models in which different faces of narcissism were tested as predictors of shyness, loneliness, and empathy.

Descriptive statistics, correlations, regression models, and exploratory factor analyses were carried out in SPSS v.24 (IBM Corp, 2016); latent class regressions models were tested in Mplus v.7.2 (Muthén and Muthén, 2012). Reliability estimates [which were assessed using McDonald’s (1999) ω total coefficient and supplemented by traditional α estimates] and parallel analysis were calculated in R (v. 3.4.2; R Development Core Team, 2016) using the *psych* package (Revelle, 2017). All of the data and syntaxes used for the purposes of the current study are freely available at: https://osf.io/gu3rs/.

## Materials and Methods

### Participants and Procedure

The study was conducted using an on-line survey. Respondents were administered a set of self-report questionnaires in Polish, and only those individuals who completed the whole set were included in the sample; thus, there were no missing observations in the sample. In total, *N* = 314 participants (67.5% female) aged between 16 and 35 years (*M* = 22.00; *SD* = 2.76) participated. Most of the participants lived in large cities (48.1%), and the rest lived in medium (14.3%) or small cities (18.8%) or in villages (18.8%). Only a few of the participants had not completed secondary education (9.2%), while the majority of participants had either completed secondary (51.9%) or higher education (38.9%). All subjects gave written informed consent in accordance with the Declaration of Helsinki. The institutional board at the Institute of Psychology, Cardinal Stefan Wyszyński University in Warsaw reviewed this project and gave us permission to implement it.

### Measures

#### Measurement of Narcissism

In assessment of grandiose narcissism, we used the Narcissistic Admiration and Rivalry Questionnaire (Back et al., 2013; Polish adaptation: Rogoza et al., 2016a), which is an alternative method to the frequently used NPI (Raskin and Hall, 1979). It comprises 18 items on which respondents answer using six-point Likert-type scales. Reliability was excellent for both admiration (ω = 0.88; α = 0.84; *M* = 3.31; *SD* = 0.84; sample item: *I show others how special I am*) and rivalry (ω = 0.88; α = 0.81; *M* = 2.71; *SD* = 0.81; sample item: *I react annoyed if another person steals the show from me*). In assessment of vulnerable narcissism, we used the HSNS (Hendin and Cheek, 1997), which comprises 10 items on which respondents answer using five-point Likert-type scales. Reliability for the scale was very good (ω = 0.80; α = 0.72; *M* = 3.31; *SD* = 0.58; sample item: *I dislike being with a group unless I know that I am appreciated by at least one of those present*).

#### Measurement of Personality

To assess the Big Five personality traits, we used the Big Five Inventory-15 (Lang et al., 2011). The scale is comprised of 15 items on which respondents answer using five-point Likert-type scales. Reliability estimates for each scale were as follows: extraversion, ω = 0.83, α = 0.81 (*M* = 2.60; *SD* = 1.00; sample item begins with: I see myself as someone who… *is outgoing, sociable*); neuroticism, ω = 0.65, α = 0.60 (*M* = 3.45; *SD* = 0.83; sample item: … *worries a lot*); openness to experience, ω = 0.77, α = 0.75 (*M* = 3.72; *SD* = 0.84; sample item: … *has an active imagination*); agreeableness, ω = 0.60, α = 0.57 (*M* = 3.32; *SD* = 0.78; sample item: … *has a forgiving nature*); and conscientiousness, ω = 0.67, α = 0.63 (*M* = 3.40; *SD* = 0.69; sample item: … *does a thorough job*). Although this inventory is designed to measure basic personality traits, it is also possible to reason about personality metatraits, which could be identified by examining the common variance of respective basic traits.

#### Measurement of Self-Esteem

The Rosenberg Self-Esteem Scale (RSES; Rosenberg, 1965; Polish adaptation: Łaguna et al., 2007) was used to measure explicit self-esteem. Respondents answer 10 items using four-point Likert-type scales. Reliability for the scale was excellent (ω = 0.93; α = 0.89; *M* = 2.65; *SD* = 0.60; sample item: *On the whole, I am satisfied with myself*). The Contingent Self-Esteem Scale (Paradise and Kernis, 1999, Unpublished) was used to measure contingent self-esteem. Respondents answer 15 items using five-point Likert-type scales. Reliability for the scale was excellent (ω = 0.88; α = 0.83; *M* = 3.41; *SD* = 0.57; sample item: *My overall feelings about myself are heavily influenced by how much other people like and accept me*).

#### Measurement of Narcissism Nomological Network

Three measures were used: the 13-item Revised Cheek and Buss Shyness Scale (Cheek and Buss, 1981; Polish adaptation: Kwiatkowska et al., 2016) measuring shyness the 20-item University of California, Los Angeles Loneliness Scale (Russell et al., 1978; Polish adaptation: Kwiatkowska et al., 2017) measuring loneliness, and the 20-item Basic Empathy Scale (Jolliffe and Farrington, 2006) measuring three components of empathy: emotional contagion, cognitive empathy, and emotional disconnection. Respondents answered all measures on five-point Likert-type scales. The reliability of each scale was as follows: shyness, ω = 0.92, α = 0.91 (*M* = 3.14; *SD* = 0.83; sample item: *I feel tense when I’m with people I don’t know well*); loneliness, ω = 0.96, α = 0.95 (*M* = 2.34; *SD* = 0.73; sample item: *There is no one I can turn to*); emotional contagion, ω = 0.86; α = 0.77 (*M* = 3.40; *SD* = 0.72; sample item: *After being with a friend who is sad about something, I usually feel sad*); cognitive empathy, ω = 0.85, α = 0.82 (*M* = 3.90; *SD* = 0.54; sample item: *I can usually realize quickly when a friend is angry*), and emotional disconnection, ω = 0.79, α = 0.72 (*M* = 2.22; *SD* = 0.66; sample item: *Other people’s feeling don’t bother me at all*).

## Results

### Mutual Relations of the Different Faces of Narcissism

First, we directly tested our preliminary expectation positing that rivalry is positively related with both admiration and vulnerable narcissism using Pearson’s correlation coefficients. It turned out that each of the analyzed relationships was significant: we observed the strongest positive relation between rivalry and vulnerable narcissism (*r* = 0.41; *p* < 0.001) and two weak relations—positive between admiration and rivalry (*r* = 0.19; *p* < 0.001) and negative between admiration and vulnerable narcissism (*r* = -0.20; *p* < 0.001). Thus, our first hypothesis was confirmed.

### Relations Between Narcissism and Personality Traits and Metatraits

To test the hypothesis on the relations between different faces of narcissism and basic personality traits, we employed linear regression models, the results of which are presented in **Table 1**.

**Table 1:** Basic personality traits predicting different faces of narcissism.

	Admiration			Rivalry			Vulnerable narcissism		
			
	*B*	*SE B*	β	*B*	*SE B*	β	*B*	*SE B*	β
Neuroticism	-0.20	0.05	-0.20^∗∗^	0.09	0.05	0.09	0.28	0.04	0.41^∗∗^
Extraversion	0.36	0.04	0.42^∗∗^	-0.04	0.04	-0.05	-0.17	0.03	-0.30^∗∗^
Openness	0.28	0.05	0.28^∗∗^	0.12	0.05	0.13^∗^	0.05	0.04	0.07
Agreeableness	-0.06	0.05	-0.05	-0.36	0.06	-0.35^∗∗^	0.00	0.04	0.00
Conscientiousness	0.16	0.05	0.13^∗∗^	-0.15	0.06	-0.13^∗^	-0.05	0.04	-0.06
*R*^2^	0.40			0.18			0.26		
*F*_(5,308)_	40.73^∗∗^			13.19^∗∗^			22.01^∗∗^		


**B* = unstandardized regression coefficient; *SE B* = standard error of the unstandardized regression coefficient; β = standardized regression coefficient. ^∗^*p* < 0.05; ^∗∗^*p* < 0.01.*

Personality traits explained 40% of the variance of admiration; extraversion and openness to experience turned out to be the strongest positive predictors, supplemented by neuroticism (negatively) and conscientiousness. Basic traits explained the least variance of the rivalry (18%), which was significantly predicted only by agreeableness (negatively). In turn, vulnerable narcissism was explained to a moderate extent (28%) by neuroticism (positively) and extraversion (negatively). Thus, the theoretical predictions regarding the pattern of relationship of the different faces of narcissism to basic personality traits, with the exception of the assumed relation between vulnerable narcissism and low agreeableness, were confirmed.

#### Metatraits Extraction

**FIGURE 3:**
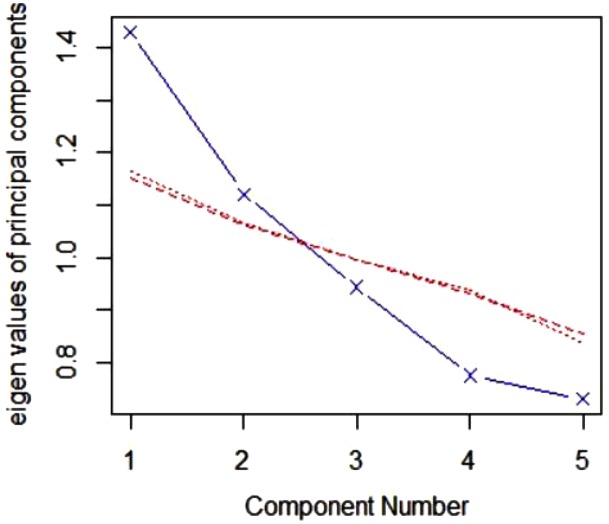
Comparison of actual and simulated eigenvalues from parallel analysis. Blue = actual data; red = simulated data.

Before assessment of the relationship between admiration, rivalry and vulnerable narcissism and personality metatraits, we conducted a parallel analysis (Horn, 1965) on five basic personality traits to assess whether personality metatraits could be meaningfully distinguished, the results of which are presented in **Figure 3**.

**FIGURE 4:**
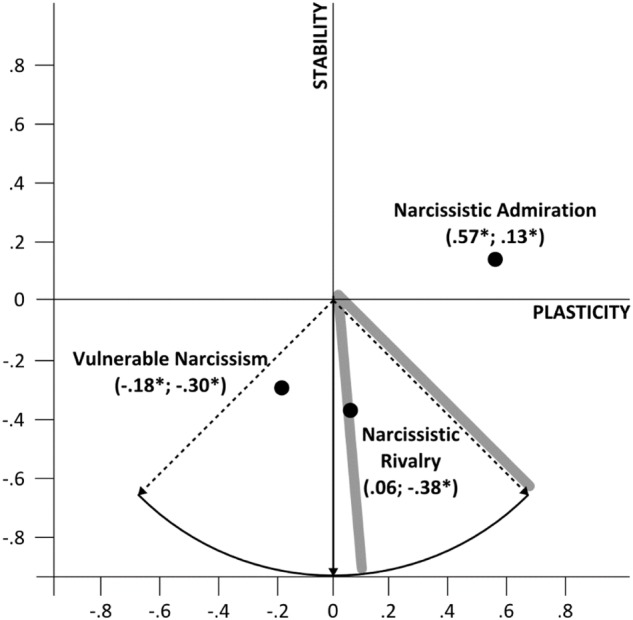
Coordinate system of personality metatraits in their relations to admiration, rivalry and vulnerable narcissism. The first value in brackets corresponds to the X-axis and the second to the Y-axis. ^∗^*p* < 0.01.

The results suggested extraction of two factors, because the eigenvalue in the actual data started to be lower in the third comparison, which reflects the theoretically predicted structure of the Plasticity and Stability metatraits in the current data. Thus, the metatraits were extracted as a first unrotated factor in an exploratory factors analysis with principal axis factoring on corresponding personality traits [i.e., extraversion and openness (factor loadings: 0.49 and 0.49, respectively) for Plasticity and neuroticism, conscientiousness and agreeableness (factor loadings: -0.46, 0.19, and 0.50, respectively) for Stability]. The distinguished metatraits turned out to be weakly correlated (*r* = 0.11; *p* = 0.048). The hypothesized relations between narcissism and personality metatraits were tested in three linear regression models, the standardized estimates of which are projected on a coordinate system (**Figure 4**), where Plasticity is the Y-axis and Stability is the X-axis.

All of the tested models were significant (*F*_(2,311)_ = 85.37; *p* < 0.001; *R*^2^ = 0.35 for admiration; *F*_(2,311)_ = 25.69; *p* < 0.001; *R*^2^ = 0.14 for rivalry; and *F*_(2,311)_ = 24.70; *p* < 0.001; *R*^2^ = 0.14 for vulnerable narcissism). Admiration and vulnerable narcissism were predicted by the Plasticity metatrait, whereas rivalry was not (*p* = 0.284). The direction of this prediction was positive for admiration and negative for vulnerable narcissism. Stability turned out to be a significant predictor of all faces of narcissism—only admiration was positively predicted, while vulnerable narcissism and rivalry were negatively predicted by Stability. Thus, the obtained results support the third hypothesis.

### Characteristics of the Different Faces of Narcissism in Individuals With Different Types of Self-Esteem

To test whether the different faces of narcissism are able to predict different types of self-esteem, a Latent Profiles Regression (LPR) was run. Contrary to the previous analyses which were variable-oriented, the LPR represents the person-oriented approach. The goal of the LPR is to test whether persons group into specific clusters with similar variable profiles and to assess if external variables are able to predict class membership. In our example, we tested whether is it possible to distinguish groups of persons with different types of self-esteem and whether different faces of narcissism predict class membership. The results of the tested latent class models differing in the number of classes are presented in **Table 2**.

**Table 2:** Results of the latent class models.

Number of classes	Class membership	BIC
1	1 = 314	3242.73
2	1 = 172	955.70
	2 = 142	
3	1 = 171	926.15
	2 = 49	
	3 = 95	
4	1 = 148	910.07
	2 = 24	
	3 = 106	
	4 = 36	
5	1 = 143	906.97
	2 = 24	
	3 = 106	
	4 = 36	
	5 = 5	
6	1 = 141	923.25
	2 = 24	
	3 = 106	
	4 = 32	
	5 = 5	
	6 = 6	


*BIC, Bayesian Information Criterion.*

**FIGURE 5:**
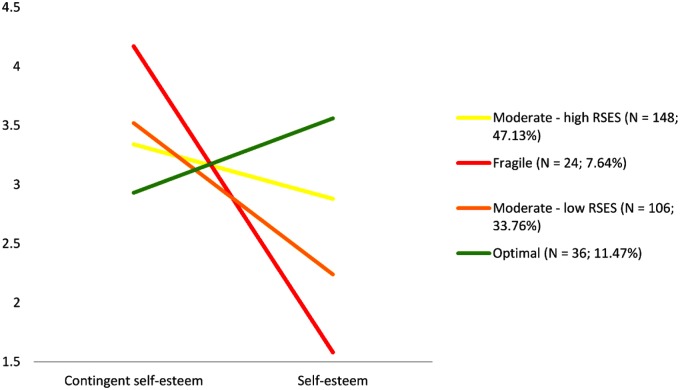
Latent profiles of individuals with different types of self-esteem. RSES = Rosenberg Self-Esteem Scale (Rosenberg, 1965).

The Bayesian Information Criterion assumed lowest value for the model with five classes suggesting its best fit to the data. However, the smallest class comprised only five persons, which makes its results difficult to interpret. Because the difference in the goodness of fit between the model with five and four classes was negligible, we maintained the latter. The mean scores in self-esteem of the model with four classes are depicted on the **Figure 5**.

The profiles of the majority of individuals representing two moderate classes are neither fragile nor optimal self-esteem. The distinction between the two moderate classes regarded only the difference in self-esteem, which was higher in the first moderate class. As expected—we also distinguished two profiles, which represented individuals with self-esteem described as fragile (combination of low self-esteem and high contingent self-esteem) and optimal (combination of high self-esteem and low contingent self-esteem). Next, we compared whether the different faces of narcissism predict membership to given class. As the LPR requires specification of one of the classes as the reference group to which results are compared, we selected the first moderate class, because it was the most numerous across the distinguished classes.

Results of the LPR presented in the **Table 3** revealed that in comparison to the moderate class with high self-esteem, all of the narcissism faces were significant predictors of class membership; however, to different extents and in different directions. Admiration was a strong negative predictor of being in a group with fragile self-esteem, negative predictor of membership in a class with moderately low self-esteem and a positive predictor of belonging to the class with optimal self-esteem. A person who scores high on rivalry is also more likely to belong to the class with fragile self-esteem and to have lower self-esteem. Interestingly, rivalry was not a significant predictor of membership in the class of individuals with optimal self-esteem. Finally, vulnerable narcissism demonstrated roughly the opposite pattern of predictions as admiration. Thus, if someone scored high on vulnerable narcissism, it was also highly probable that he or she would have fragile self-esteem; moderate scores predict belonging to the moderate class, although with an associated lower level of self-esteem. Having a low score on vulnerable narcissism is a significant predictor of having optimal self-esteem, as well. All in all, the formulated hypotheses regarding the predominance of different faces of narcissism in individuals with different types of self-esteem were confirmed.

**Table 3:** Coefficients from Latent Profiles Regressions.

	Fragile		Moderate (low RSES)		Optimal	
			
	*B*	*SE B*	*B*	*SE B*	*B*	*SE B*
Admiration	-5.07^∗∗^	1.25	-2.90^∗∗^	0.65	2.33^∗∗^	0.60
Rivalry	1.75^∗∗^	0.60	0.98^∗^	0.46	-0.04	0.48
Vulnerable narcissism	4.20^∗∗^	1.33	2.21^∗^	1.02	-1.31^∗^	0.62


**B* = unstandardized regression coefficient; *SE B* = standard error of the unstandardized regression coefficient. The moderate class [high RSES = Rosenberg Self-Esteem Scale (Rosenberg, 1965)] was chosen as the reference group. ^∗^*p* < 0.05; ^∗∗^*p* < 0.01.*

### Nomological Network

To test the last hypothesis regarding the nomological network of the different faces of narcissism, we investigated their associations with shyness, loneliness, and empathy. The standardized estimates obtained from linear regression models are presented in **Table 4**.

**Table 4:** Different faces of narcissism predicting shyness, loneliness, and empathy.

	Shyness			Loneliness			Emotional contagion			Cognitive empathy			Emotional disconnection		
					
	*B*	*SE B*	β	*B*	*SE B*	β	*B*	*SE B*	β	*B*	*SE B*	β	*B*	*SE B*	β
Admiration	-0.56	0.04	-0.57*	-0.35	0.04	-0.41**	0.04	0.05	0.04	0.15	0.04	0.23**	0.02	0.04	0.03
Rivalry	0.09	0.04	0.09*	0.15	0.05	0.16**	-0.32	0.05	-0.35**	-0.25	0.04	-0.37**	0.34	0.05	0.42**
Vulnerable narcissism	0.57	0.06	0.40**	0.44	0.07	0.34**	0.49	0.08	0.39**	0.02	0.06	0.03	-0.12	0.07	-0.10
*F*_(3,310)_	143.67^∗^			65.50^∗^			18.68^∗^			17.97^∗^			19.12^∗^		
*R^2^*	0.58			0.39			0.15			0.15			0.16		


**B* = unstandardized regression coefficient; *SE B* = standard error of the unstandardized regression coefficient; β = standardized regression coefficient. ^∗^*p* < 0.05; ^∗∗^*p* < 0.01.*

In general, narcissism explained the most variance in shyness and loneliness, respectively, and only a modest amount of variance in empathy. In regard to shyness and loneliness, admiration and vulnerable narcissism were contradictory predictors, i.e., admiration as a negative and vulnerable narcissism as a positive predictor. Rivalry turned out to be the strongest negative predictor of emotional contagion and cognitive empathy, and strongest positive predictor of emotional disconnection. Admiration predicted only cognitive empathy, while vulnerable narcissism predicted only emotional contagion. Summarizing, the relations of the different faces of narcissism assumed the theoretically predicted pattern, and thus, the last of the hypotheses was also confirmed.

## Discussion

The current study aimed to test the dimensions distinguished within the NSM (Krizan and Herlache, 2017) in an empirical setting in regard to their relations with other personality traits, self-esteem, and the nomological network of shyness, loneliness, and empathy. Because other empirical studies pointed out that vulnerability and grandiosity are orthogonal dimensions (e.g., Wink, 1991), our main expectation regarded the self-importance dimension (as most represented by the narcissistic rivalry) will be positively associated with both vulnerability and grandiosity. As an initial check, we directly tested if this assumed relation exists, as partially suggested by previous studies (e.g., Miller et al., 2014). This hypothesis was confirmed early in the study: whereas vulnerable narcissism and admiration were negatively related to each other, they both were positively related to narcissistic rivalry. Thus, it may be claimed that the self-importance dimension of the NSM indeed may be suggested to be the core of narcissism, as it links the orthogonal dimensions of vulnerability and grandiosity (Krizan and Herlache, 2017).

To better understand this observed relationship, we investigated how the different faces of narcissism are related to basic personality traits and personality metatraits. The relationships of both types of narcissism to basic personality traits were mostly replications of the results from previous studies (e.g., Hendin and Cheek, 1997; Rogoza et al., 2016b; Leckelt et al., 2018): vulnerable narcissism was predicted by high neuroticism and low extraversion, rivalry was predicted by low agreeableness, and admiration was predicted by high extraversion and openness to experience. Whereas our results are in line with the work of Miller et al. (2017b), who point out that vulnerable narcissism is mostly a disorder of neuroticism, we did not find any relations with agreeableness, which as should be the latter in strength correlate. In the current study, we found that low extraversion was the important predictor of vulnerable narcissism, which also is reported within the literature (e.g., Miller et al., 2014). According to the NSM (Krizan and Herlache, 2017), it appears that our conclusions concerning vulnerable narcissism are only applicable to the marginal border of the vulnerability dimension, which is negatively related to the grandiosity dimension, whereas Miller et al. (2017b) referred to the more central aspects of vulnerable narcissism, which are closely related to the self-importance dimension.

Previous studies have found that admiration was primarily associated with high Plasticity and rivalry with low Stability (Rogoza et al., 2016c). These relationships were also found in the current study. The current study was the first to examine the associations between vulnerable narcissism and personality metatraits. The results suggest that vulnerable narcissism is related to low Plasticity and low Stability, which seems to support the hypothesis that the dimensions of the NSM and the metatraits of personality empirically overlap, i.e., that Plasticity and Stability are highly associated with different faces of narcissism, with Plasticity typically being low for vulnerability and high for grandiosity, whereas low Stability is characteristic of both vulnerable and grandiose narcissism. Krizan and Herlache (2017) argued that the vulnerable and grandiose dimensions are also associated with differences in temperament, i.e., avoidant for vulnerable and approaching for grandiose narcissism. Plasticity is strongly associated with temperament among narcissists; when Plasticity is low, temperament is most likely to be avoiding and when Plasticity is high, the temperament is most likely to be approaching (Strus and Cieciuch, 2017). The current results support these conclusions as low Stability turned out to be the common core of narcissism, whereas the Plasticity differentiated vulnerability from grandiosity. Thus, it may be concluded that it is possible to interpret narcissism within the broader framework of the Two Factor Model of personality (Cieciuch and Strus, 2017).

In the current study, we not only focused on explaining the relationships between variables, but also assessed how the participants differed among themselves. For this purpose, we investigated how the different faces of narcissism predict falling into different categories of self-esteem. Four classes were differentiated: the least numerous classes comprised individuals whose self-esteem is either fragile or optimal, whereas individuals whose self-esteem was neither fragile nor optimal made up the majority of the studied sample (albeit it was divided in two subgroups with high and low self-esteem). The different faces of narcissism were used as predictors of membership in each class. Admiration is described as the bright face of narcissism (Back et al., 2013; Rogoza et al., 2016b); thus, higher scores on admiration in individuals whose self-esteem was optimal and higher than average were expected and subsequently confirmed. Admiration thus seems to be the functional strategy of narcissism, allowing for adaptation designed to deal with the costs produced by the dark face of narcissism—rivalry (Back et al., 2013; Leckelt et al., 2015; Rogoza et al., 2018). The dark side of narcissism did not vary strongly across the distinguished classes, although it was slightly elevated in the classes with lower self-esteem. These results corroborate the findings of Wetzel et al. (2016) who reported that it is possible to distinguish class which scores higher on admiration but not on the rivalry. Finally, individuals with fragile self-esteem, which is most strongly associated with depression-proneness and anxiety (Kernis, 2003), were most likely to also score high in vulnerable narcissism. As this face of narcissism is also associated with negative affect, psychopathology (e.g., anxiety, depression, paranoia), and difficulties in the therapeutic relationship (Cain et al., 2008; Miller et al., 2017b), it might be stated that vulnerable narcissism is actually the blue face of narcissism.

The nomological network of the different faces of narcissism with regard to shyness, loneliness, and empathy was also assessed. Results obtained in the current study mostly replicate existing results (e.g., Hendin and Cheek, 1997; Back et al., 2013; Fatfouta, 2017); however they are presented in a more systematical manner. We revealed a contradictory pattern of associations between the bright and blue face of narcissism with regard to shyness and loneliness (negative for the bright and positive for the blue face of narcissism). These results corroborate existing reports, as both shyness and loneliness are related to low Plasticity and low Stability and, moreover, with anxiety-related disorders (e.g., social anxiety disorder; Kwiatkowska et al., 2016; Poole et al., 2017). The dark side of narcissism was predominantly related to low empathy, which underpins its antagonistic and socially exploitative character (Leckelt et al., 2015). Finally, we found that admiration was positively linked with cognitive empathy, which may be the result of higher level of social desirability (Kowalski et al., 2018), whilst vulnerable narcissism was positively linked with emotional contagion, which might be explained by the heightened levels of neuroticism (Miller et al., 2017b).

Summarizing, our findings suggests that the NSM (Krizan and Herlache, 2017) was successfully reproduced in an empirical setting and the location of its dimensions was confirmed in the broader context of the Two Factor model of personality metatraits (Cieciuch and Strus, 2017). We confirmed that rivalry is central to both the vulnerability and grandiosity dimensions of narcissism. Also, our results pointed out that vulnerable narcissism may be seen as the blue face of narcissism, which supplements the existing differentiation of the bright and dark faces of narcissism.

## Author Contributions

RR drafted the paper; MŻ-P critically reviewed the paper; MK and KK made literature review and assisted in analyses.

## Conflict of Interest Statement

The authors declare that the research was conducted in the absence of any commercial or financial relationships that could be construed as a potential conflict of interest.
